# A well-perceived, blind image quality assessment algorithm using an enhanced noise feature criterion

**DOI:** 10.1038/s41598-026-54147-2

**Published:** 2026-05-23

**Authors:** Yi-Pin Hsu, Chuan-Yen Hsiao

**Affiliations:** Department of Computer Science and Information Engineering, Ming Chuan University, Taoyuan Campus - No. 5, Deming Rd., Guishan District, Taoyuan, Taiwan 333

**Keywords:** Blind image quality assessment, Perception-based noise feature criterion, No-reference image quality assessment method, Immediate application, Engineering, Mathematics and computing

## Abstract

Many deep learning-based blind image quality assessment (BIQA) methods achieve high accuracy but rely heavily on complex network architectures and large datasets, which limit their applicability. This study proposes an enhanced perception-based no-reference (NR) BIQA method that incorporates a revised noise feature criterion for immediate and practical use. This approach was motivated by observations that conventional noise feature analysis becomes unstable in images with strong horizontal structures, such as fence-like patterns. To address this limitation, improved noise weighting and decision criteria were introduced. The method was evaluated on four publicly available databases (LIVE, CSIQ, TID2013, and KADID-10k), demonstrating higher or comparable prediction performance relative to the baseline algorithm, as measured by Spearman rank order correlation coefficient (SROCC) and Pearson linear correlation coefficient (PLCC). A detailed comparative analysis of quality estimation performance was conducted between the reference algorithm and the proposed algorithm. The estimated image quality scores were presented side by side, demonstrating that the proposed algorithm achieved more accurate estimations for the 24 perfect and distortion-free images in the TID2013 dataset. The results showed that the proposed algorithm placed all images closer to the ‘Excellent’ quality region according to Matlab help center description, aligning more closely with the expected evaluation goals than the reference algorithm.

## Introduction

Research and applications commonly begin with input source verification, as variations in source quality influence algorithm design or system evaluation. In video processing, low-quality frames are encoded with smaller quantization parameters to preserve quality for subsequent reference during intra-frame processing^1,2^. Recent studies have focused on developing optimal rate control mechanisms by correlating image and video quality assessments with coding rates^3^. Objective image quality assessment (IQA) has been introduced to provide quality predictions aligned with subjective perception. IQA methods are generally categorized into three types: full-reference^4–6^, reduced-reference^7–9^, and no-reference (NR)^10–16^ or blind IQA (BIQA). NR methods are widely studied due to their applicability without auxiliary data or additional memory requirements. This study builds upon the characteristics of NR IQA to propose an improved approach.

BIQA is used to estimate image quality scores without requiring original references or auxiliary data. Earlier approaches primarily relied on predefined distortion-specific models, introducing degradations, such as blur^17,18^, noise^19^, JPEG compression^20^, or contrast adjustment^21,22^. For instance, phase congruency and gradient magnitude features were extracted in^17^ to quantify blur severity in out-of-focus images. A recent study^24^ introduced an NR quality metric for camera-captured images, incorporating low-level features (brightness, saturation, contrast, sharpness, naturalness) and high-level semantic descriptors, with support vector regression used for quality prediction. Similarly^25^, emphasized perceptual features derived from fundamental image processing operations, enabling immediate application across domains. However, distortion-specific methods remain limited in scope, as they depend on prior knowledge of noise or artifact types. General-purpose BIQA, in contrast, is developed to evaluate image quality without assumptions about underlying distortions.

BIQA approaches are categorized into supervised and unsupervised methods. From the learning-based concept, supervised methods rely on subjective scores as ground truth to train prediction models. Their development primarily differs in feature extraction and regression strategies. In^11^, statistical features were extracted from the wavelet domain and mapped to quality scores using a support vector machine. In^10^, natural scene statistics were selected to represent image quality effectively, while^23^ combined DCT and wavelet-based features with label transformation for prediction. Convolutional neural networks^26–28^ have recently been adopted, demonstrating strong performance in IQA but requiring substantial computational resources during training and frequent retraining when applied to new domains. Although supervised BIQA methods achieve high prediction accuracy, their reliance on large-scale databases and high training costs limits their practicality for rapid or real-time quality estimation.

Unsupervised BIQA methods estimate image quality without relying on subjective scores and have demonstrated performance comparable to supervised approaches. In^12^, the natural image quality evaluator (NIQE) was developed by modeling mean-subtracted contrast normalized (MSCN) coefficients using generalized and asymmetric Gaussian distributions, with quality-aware features extracted from paired MSCN products. In^29^, the local NIQE (IL-NIQE) method was improved through the inclusion of gradient features, Log-Gabor filter responses, and color information. In^30^, local binary pattern statistics were utilized to compute a local binary pattern index (LPSI) for quality estimation. In^30^, the perception-based image quality evaluator (PIQE) was introduced as a training-free method that assesses distortion through local feature extraction and quality mapping functions, with predictions derived from block-level image characteristics.

The concepts underlying NIQE, IL-NIQE, LPSI, and PIQE emphasize naturalness and structural features, which provide image quality estimation comparable to supervised BIQA methods and are suitable for practical applications. Although NIQE, IL-NIQE, and LPSI operate without training, they still rely on partial statistical processing, limiting their generalization capabilities. Extensive research evaluations have validated their effectiveness, leading to the inclusion of learning-based NIQE and structure-based PIQE in MATLAB’s NR IQA toolbox.

As PIQE lacks training capabilities for real-time or immediate applications, this study introduces an enhanced BIQA method, termed enhanced PIQE (EPIQE), built upon the PIQE framework to provide a more comprehensive assessment of image quality. The proposed method focuses on refining perception-based noise feature criteria to achieve robust structural evaluation for widely used IQA tasks. The contributions in this work as twofold: (1) EPIQE incorporates a revised perception-based noise feature criterion equation and demonstrates superior performance to PIQE across extensive evaluations using open natural image databases; (2) a detailed comparison of PIQE and EPIQE prediction accuracy is conducted using 24 reference images from TID2013 without distortion, serving as ground truth, to compare the results of estimated scores closer to the excellent quality range.

The paper is organized as follows: first, the architecture of PIQE is briefly reviewed, with key components highlighted. Next, tests on real images are used to identify the limitation of PIQE, specifically a defect in the perception-based noise feature criterion. Finally, three open databases, including LIVE, TID2013, and CSIQ, are employed to validate IQA performance and compare PIQE and EPIQE predictions, using distortion-free reference images from TID2013 as ground truth. The evaluation demonstrates that EPIQE consistently outperforms PIQE across all three databases and effectively addresses a wider range of image conditions while maintaining generalization.

## Methodology

### Brief review of the PIQE algorithm

The PIQE algorithm differs from conventional geometric structure-based approaches by emphasizing human visual attention to highlight salient image regions or active spatial areas. Local quality assessments at the block or patch level contribute to the overall perceived image quality, with experiments indicating that distortion estimation is best performed on 16 ⋅ 16 blocks^30^. The algorithm involves two primary steps: divisive normalization applied to the input image and local mean removal as part of preprocessing. Block-level distortion estimation is then conducted based on two criteria: noticeable distortion and noise features. The results from these criteria are combined to calculate block scores using the PIQE definition equation.

#### Divisive normalization

In^32^, natural scene statistics features extracted from grayscale images were utilized to remove the local mean and apply divisive normalization. This process is defined for the input luminance image $\:I\left(i,j\right)$ and output luminance image $\:\widehat{I}\left(i,j\right)$ as follows:

1 $$\:\widehat{I}\left(i,j\right)=\frac{I\left(i,j\right)-\mu\:(i,j)}{\sigma\:\left(i,j\right)+1}$$

where *(i*,* j)* represents spatial indices ranging from 1 to the image height and width boundaries, respectively. The residual is computed using (2) and (3), where a window size of 3 is applied for the operation.

2 $$\:\mu\:\left(i,j\right)={\sum\:}_{k=-3}^{3}{\sum\:}_{l=-3}^{3}{\omega\:}_{k,l}{I}_{k,l}(i,j)$$

3 $$\:\sigma\:\left(i,j\right)=\sqrt{{\sum\:}_{k=-3}^{3}\sum\:_{l=-3}^{3}{\omega\:}_{k,l}{\left({I}_{k,l}\left(i,j\right)-\mu\:(i,j)\right)}^{2}}$$

The results of (1) are referred to as MSCN coefficients, with a detailed explanation provided in^10^. As indicated in (1), the image is divided into non-overlapping blocks, and the variance of each block is computed. Due to the normalization process, the variance of the normalized coefficients is constrained between 0 and 1. A threshold of 0.1 is recommended in^31^ for identifying non-uniform blocks, as this range is considered reasonable. Blocks with variance values greater than 0.1 are classified as non-uniform or spatially active.

#### Block level distortion estimation

A common form of image distortion involves quantization, which appears as blurring or the addition of noise. These effects are simultaneously evaluated using two metrics: the noticeable distortion criterion and the noise feature criterion.

##### Noticeable distortion criterion

In^33^, an edge segment is defined as a set of six contiguous pixels, with four edge segments considered around each block boundary. A block is classified as an active spatial block if the standard deviation $\:{\sigma\:}_{seg}$ of all segment values within this collection is below a specified threshold. As recommended in^31^, a threshold of 0.1 is used based on experimental results. The generalized formulation for this condition is presented in (4).

4 $$\:{\sigma\:}_{seg}<0.1$$

##### Noise feature criterion

**Fig. 1:**
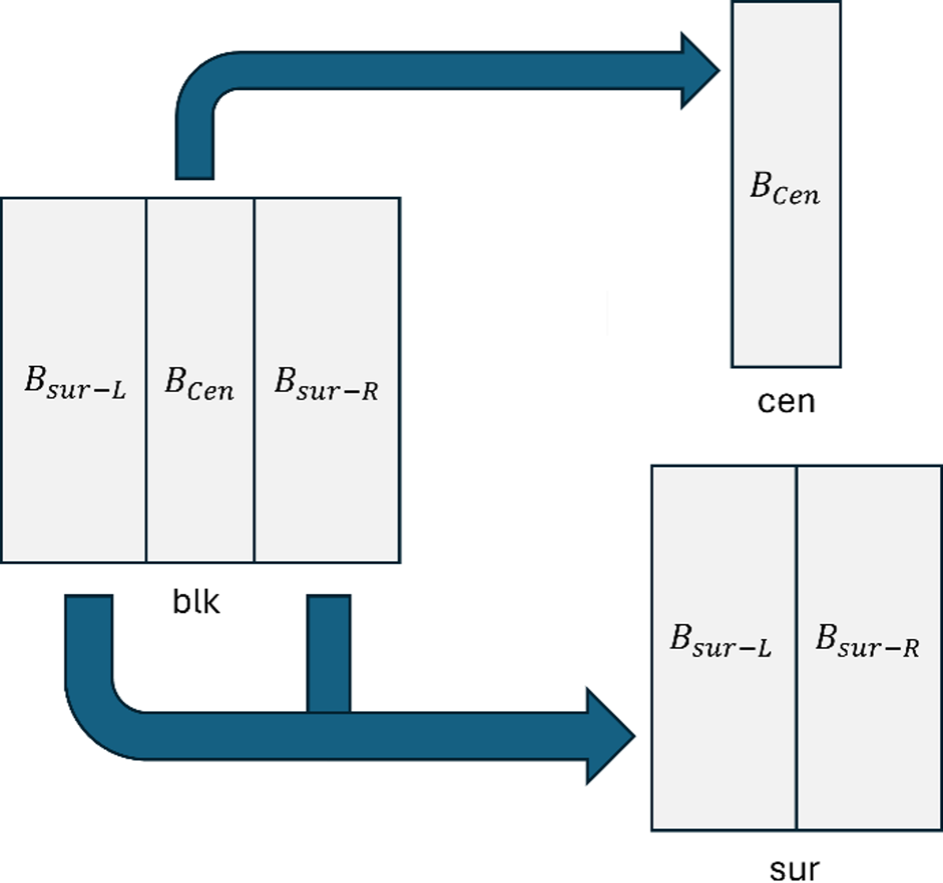
Center (cen by $\:{B}_{cen}$) and surround (sur by $\:{B}_{sur-L}$ and $\:{B}_{sur-R}$) regions of a block.

Since image quality is determined by the human visual perception system, a perception-based center-surround criterion is employed to estimate noise distortion at the block level using MSCN features. This criterion is derived from the human visual system’s sensitivity to center-surround variations. A widely recognized approach for measuring this sensitivity to variance analysis, where block-level variance is used as the noise feature criterion. Each block is divided into two regions (Fig. 1). One is the central region, containing the two center columns, $\:{B}_{cen}$ and the surrounding region $\:{B}_{sur}$, composed of the remaining two columns $\:{B}_{sur-L}$ and $\:{B}_{sur-R}$.

Furthermore, to formulate the perception equation, an empirical relationship is established between the center-surround deviation ratio and the block standard deviation of the corresponding MSCN block, as expressed in (5):

5 $$\:\beta\:=\frac{\left|\left(\frac{{\sigma\:}_{Cen}}{{\sigma\:}_{sur}}\right)-{\sigma\:}_{blk}\right|}{max\left(\left(\frac{{\sigma\:}_{Cen}}{{\sigma\:}_{sur}}\right),{\sigma\:}_{blk}\right)}$$

where $\:{\sigma\:}_{Cen}$ and $\:{\sigma\:}_{sur}$ denote the standard deviations of the central $\:{B}_{cen}$and surrounding segment $\:{B}_{sur}$, respectively, and $\:{\sigma\:}_{blk}$ represents the standard deviation of the spatially active block as defined in (1). A block $\:{B}_{k}$ is classified as noise-affected if its computed value $\:{\sigma\:}_{blk}$ exceeds a specified threshold. The empirical relationship and generalized formulation for this condition are provided in (6):

6 $$\:{\sigma\:}_{blk}>2*\beta\:$$

The final image quality score, $\:{PIQE}_{score}$, is defined in (8), where $\:{N}_{SA}$ denotes the number of spatially active blocks in the image, $\:{D}_{sk}$ represents the distortion measure, and $\:{v}_{blk}$ indicates the variance for each distorted block under varying conditions as determined by (6)–(8):

7 $$\:{D}_{sk}=\left\{\begin{array}{c}1,\:\:if\left(4\right)\:and\:\left(6\right)\:are\:true\\\:{v}_{blk},\:\:if\left(6\right)\:is\:ture\\\:{(1-v}_{blk)},\:\:if\left(4\right)\:is\:true\end{array}\right.$$

8 $$\:PIQ{E}_{Score}=\frac{\left({\sum\:}_{k=1}^{{N}_{SA}}{D}_{sk}\right)+1}{{N}_{SA}+1}$$

From the above description, the calculation of $\:{PIQE}_{score}$ is based on (6)–(8) to determine the corresponding block variance, with values ranging from 0 to 100. A score of 100 represents poor image quality, whereas lower values indicate better quality.

### Observation and pattern analyses

**Fig. 2:**
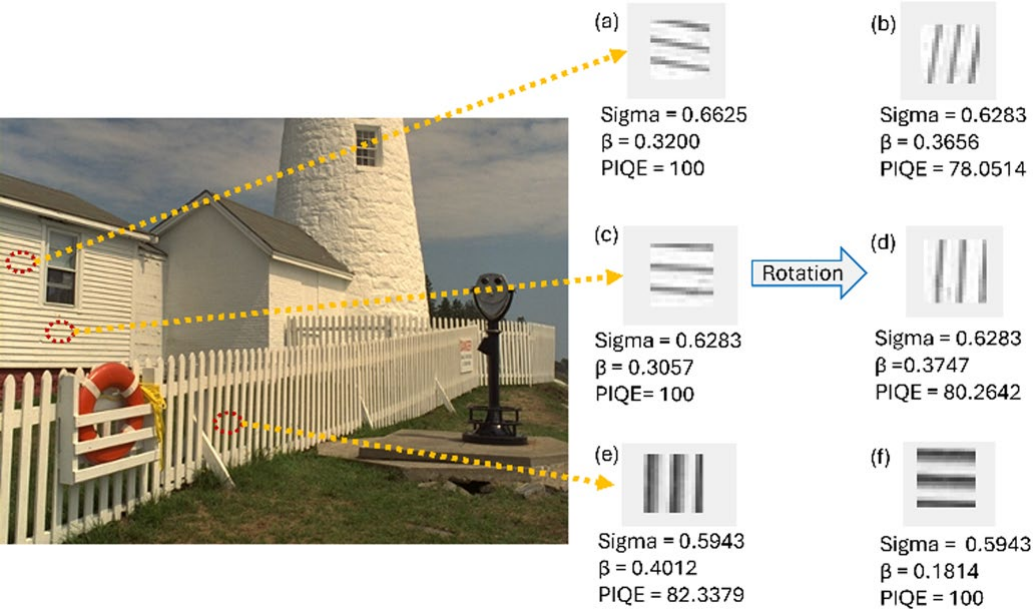
Standard deviation (*σ)*, *β* value, and PIQE score of three blocks emphasizing horizontal and vertical shapes.

The primary goal of BIQA methods^31^ is to assess image quality without the need for a reference image, which is essential in full-reference approaches. The MSCN coefficients from (1) are recommended for further processing. Without relying on learning-based strategies and with a focus on real-time applications, a perception-driven approach is employed to minimize distortions caused by geometric structures. Numerous statistical models^24–28^ have been developed to approximate distortions and estimate image quality; however, due to the variability of real-world image conditions, achieving a balance between accuracy and computational efficiency remains challenging, resulting in disproportionate costs. Leveraging human visual system-based perception principles, PIQE has demonstrated strong performance in terms of accuracy, usability, and generalization. A critical component of this approach is the noise feature criterion, integrated into the perception-based structure design (Fig. 1). Nevertheless, a limitation of the vertical-direction structure is its reliance on the comparison between the standard deviation of a segment and that of the corresponding block, as defined in (5) and (6). To examine the impact of this limitation, Fig. 2 presents an example using an image from the TID2013 database, where the standard deviation, *β* (from 5), and PIQE scores (from 8) are evaluated across three different blocks.

Three 16 × 16 pixel blocks, labeled (a), (c), and (e), are first cropped from the image, and their standard deviation (*σ)*, β values (from 5), and PIQE scores are calculated. Block (b) is obtained by rotating block (a) by 90 degrees, while blocks (d) and (f) are similarly derived from blocks (c) and (e), respectively. The comparison reveals a clear drawback: although the standard deviation remains unchanged, the *β* values and PIQE scores differ between (a) and (b), as well as between (c) and (d). Furthermore, block (e), originally extracted from the image in a vertical orientation, exhibits variations in *β* values when rotated, as seen in block (f). This leads to a PIQE score reaching the maximum value of 100, calculated based on an incorrect *β* value. These observations indicate a structural flaw in (5), where image quality estimation is affected when block content features horizontal patterns. The block rotation analysis supports the assumption that both vertical and horizontal orientations must be considered. In summary, efficient detection of block orientation and appropriate adjustment of *β* in (5) are necessary for improved estimation accuracy.

**Fig. 3:**
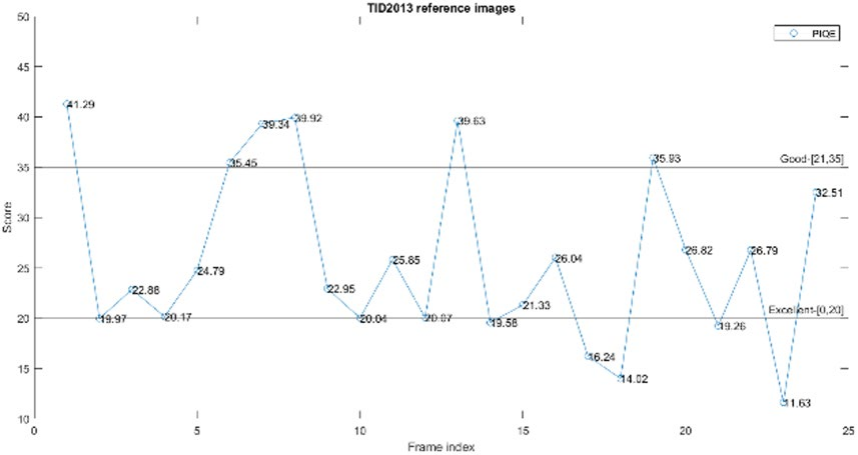
PIQE scores of 24 reference images from the TID2013 database.

The PIQE score was also evaluated using MATLAB’s built-in function, calling piqe(), to obtain mapped scores for comparison with reference images. The TID2013 database provides original, distortion-free images for this evaluation. According to the MATLAB help center, PIQE categorizes image quality into five levels: Excellent (0–20), Good (21–35), Fair (36–50), Poor (51–80), and Bad (81–100). Among all reference images, only four are classified as Good and three as Excellent, while the majority fall within the Fair category (Fig. 3). However, visual inspection of these distortion-free images shows that they are clear and visually pleasing, suggesting that their scores should be near the Excellent range. Therefore, the proposed EPIQE method aims to produce more accurate quality scores, with all reference images expected to be closer to the Excellent category.

The fence-like pattern observed in image content is evident when reviewing the four images with the highest PIQE scores from the previous analysis. This observation supports the deduction that the influence of such patterns should be minimized to ensure more accurate image quality score estimation.

### Correction of perception-based noise feature criterion

To address the impact of horizontal fence patterns and improve perception-based noise feature estimation, the strategy is proposed to enhance PIQE accuracy. An efficient preprocessing rule is introduced to eliminate the influence of horizontal fence shape patterns during block analysis. Then, (5) is modified to provide a more accurate representation of noise features.

The horizontal fence pattern, as a strong edge feature, enables block-level preprocessing to effectively eliminate flat noise, thereby reducing false detections in complex image content. Smoothing filters are widely applied for basic noise suppression due to their simplicity and effectiveness^34^. In this study, Gaussian filtering is employed for each block-level flat noise removal, as defined in (9):

9 $$\:G\left(x,y\right)=\frac{1}{2\pi\:{\sigma\:}^{2}}{e}^{\raisebox{1ex}{$-({x}^{2}+{y}^{2})$}\!\left/\:\!\raisebox{-1ex}{$2{\sigma\:}^{2}$}\right.}$$

where *\:G(x,y)* represents the Gaussian mask at coordinates x and y, and *σ* denotes the standard deviation parameter of the Gaussian function. A larger *σ* value increases the smoothing effect. Conversely, a smaller σ value preserves more image texture, similar to Canny, thus negating the function of using this filter, with σ = 4 recommended based on empirical evaluation.

In general, smoothing is performed by convolving the original block *\:blk(x,y)* of size (h × w) with a Gaussian mask *\:G(i,j)* as shown in (10). The process involves calculating the sum of products between the input block and a smaller Gaussian kernel of size 3 × 3. The resulting block $\:{blk}^{s}$, obtained after Gaussian filtering, has its flat noise effectively removed and is prepared for subsequent horizontal fence pattern detection.

10 $$\:{blk}^{s}(x,y)=\sum\:_{i=0}^{h-1}\sum\:_{j=0}^{w-1}G(i,j)blk(x-i,y-j)$$

In the next step, the objective is to detect the presence of horizontal fence patterns, as this detection directly influences the determination of the new *β* value. After applying (9), edge features become more prominent, making the identification of horizontal fence structures more accurate.

The Sobel operator is adopted as an effective and computationally simple method for detecting horizontal fence patterns. It provides approximate gradient values and identifies edge directions without requiring contextual information. In digital image processing, edge detection is commonly performed using convolution between an image and a kernel or mask^34^. The Sobel method uses a gradient-based approach that searches for strong first-derivative changes, which align with the need to identify horizontal fence structures. By measuring the 2D spatial gradient within each block, the operator emphasizes high-spatial-frequency regions corresponding to edges. Consequently, the Sobel detector is recommended for horizontal fence shape detection.

**Fig. 4:**
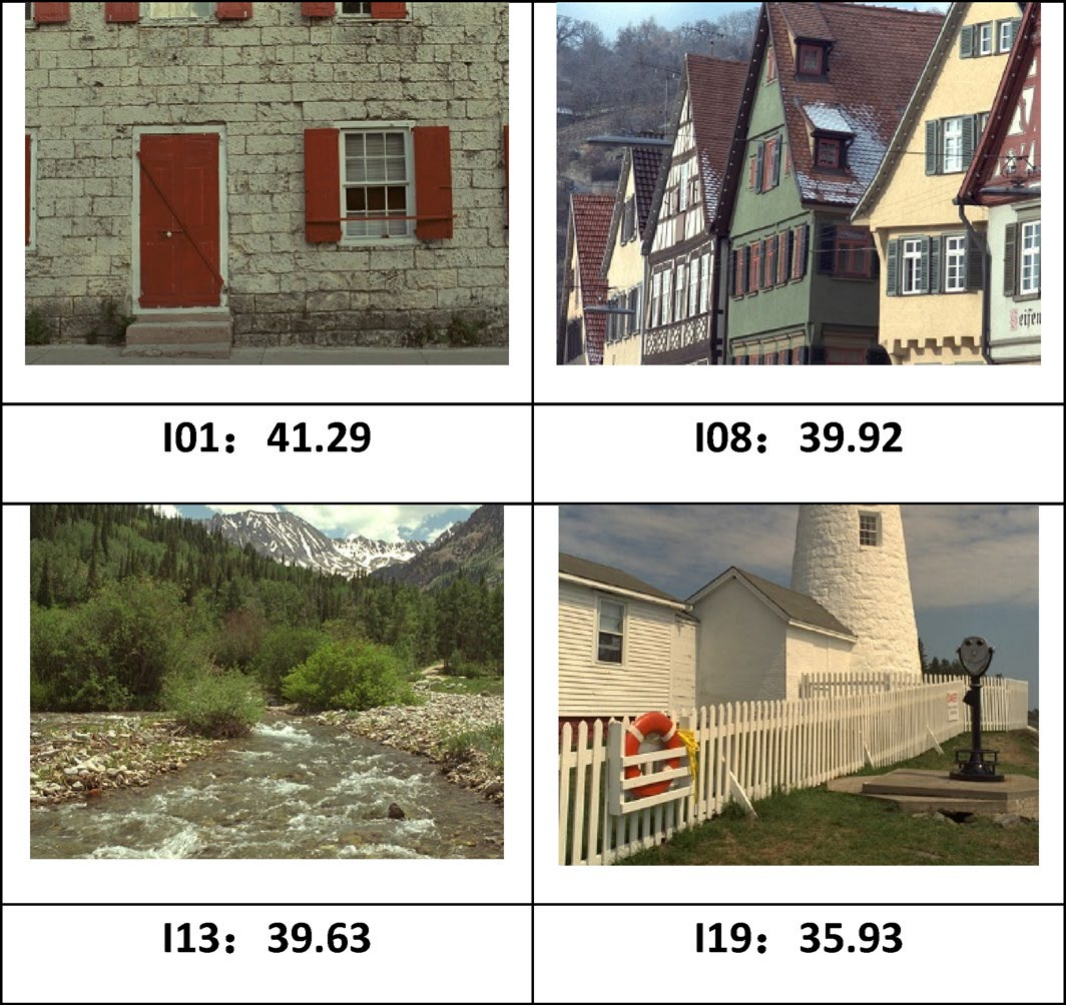
Edge detection results using Canny and Sobel filters for image I19.bmp from TID2013: (**a**) The *\:blk* extraction at position (140, 430); (**b**) The *\:blk* extraction at position (200, 200).

The results of applying Sobel and Canny edge detectors to *\:blk*, obtained after Gaussian smoothing, are demonstrated (Fig. 4). The Canny detector captures a greater amount of image content compared to the Sobel detector; however, this outcome is undesirable for the intended task, as it introduces errors in horizontal fence pattern detection. In contrast, the Sobel detector successfully avoids detecting irrelevant content, making it a more suitable choice for accurate horizontal fence shape detection.

**Fig. 5:**
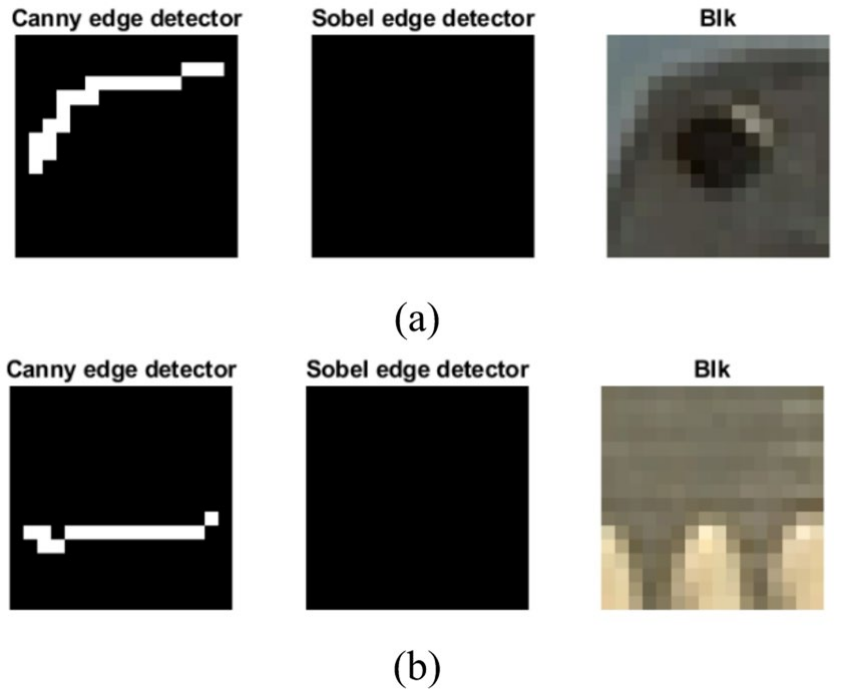
The flowchart for calculating the PIQE score calculation of the entire image.

**Fig. 6:**
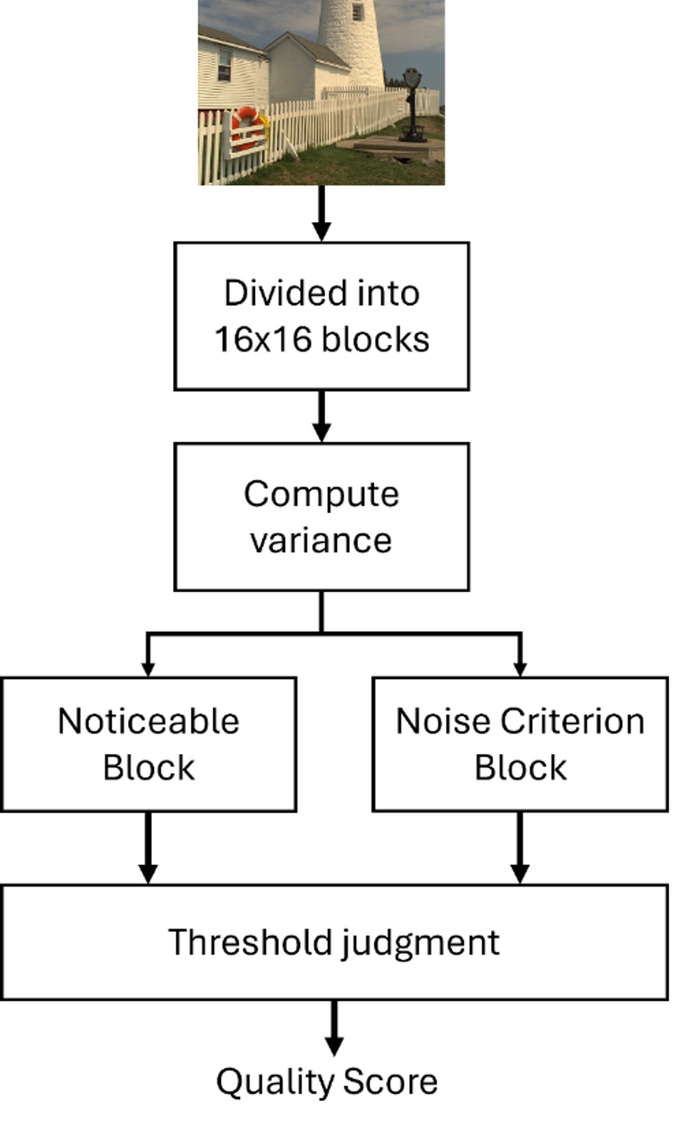
The flowchart of noiseCriterion for blockSigma and blockBeta calculation.

Based on the edge detection results, a formula was developed to calculate the sum of peak values in both vertical and horizontal directions, as expressed in (11) and (12). The maximum value γ is then obtained using (13) from the collection of $\:{blk}_{v-seg}\left(y\right)$ and $\:{blk}_{h-seg}\left(x\right)$. A threshold of γ > 4, determined empirically, is used to decide whether a horizontal fence-shaped edge is present. If such an edge is detected, (5) is modified and replaced with the updated formulations in (14)–(15). Otherwise, (5) remains valid for image quality score computation. A threshold that is too low may misinterpret normal images as vertical or horizontal fan-shaped features.

11 $$\:{blk}_{v-seg}\left(y\right)=\sum\:_{i=0}^{h-1}{blk}^{s}(i,y)$$

12 $$\:{blk}_{h-seg}\left(x\right)=\sum\:_{j=0}^{w-1}{blk}^{s}(x,j)$$

13 $$\:\gamma \: = \arg \mathop {\max }\limits_{{x,\:\:y \in \{ 1, \ldots \:,N\} }} \left( {blk_{{h - seg}} \left( x \right),\:blk_{{v - seg}} \left( y \right)} \right)$$

14 $$\:\beta\:=\frac{\left|\alpha\:-{\sigma\:}_{blk}\right|}{max\left(\alpha\:,{\sigma\:}_{blk}\right)}$$

15 $$\:\alpha \: = \arg \mathop {\max }\limits_{{B \in \{ blk,\:\:\:blk^{t} \} }} \left( {\frac{{\sigma \:_{{Cen}} \left( B \right)}}{{\sigma \:_{{sur}} \left( B \right)}}} \right)$$

where $\:{blk}^{t}$ denotes *\:blk* the transpose operation, while *w* and *h* represent the block width and height, respectively. Following the PIQE recommendation, a block size of *w* × *h* = 16 × 16 is used in the experiments. Since the block is rectangular, *w* = *h* = 16, resulting in *N* = 16. If the Gaussian filter is not used for processing, and instead the number of vertical and horizontal stripes is calculated directly using (11)-(12), the random distribution characteristics of the image texture will make it hard to select a suitable r value, and thus it will be hard to correct (15). The flowchart in Fig. 5 for calculating the PIQE score of the entire image is shown below, where blockImpaired and “blockSigma, blockBeta” are obtained by two functions, respectively. The flowchart of noiseCriterion in Fig. 6 for blockSigma and blockBeta are shown below. The correction proposed in this study is the calculation of blockBeta, which is (14).

The execution steps are as follows:

Step 1: Perform calculations using a Gaussian filter (10) to obtain the output.

Step 2: Perform edge detection using ‘Sobel’ on the result of the previous step to obtain the output.

Step 3: Calculate the number of horizontal and vertical blocks using (11) and (12).

Step 4: Calculate the maximum value ‘r’ using (13).

Step 5: If *r* > 4, use (14) and (15) to obtain the result, which is blockBeta. Otherwise, use the original PIQE blockBeta calculation method.

## Experimental results

This section presents the performance evaluation of the proposed BIQA method, including the experimental setup, a comprehensive comparison across datasets with various distortion types, and a detailed accuracy analysis of PIQE versus EPIQE, focusing on applicability without training.

### Experimental guideline and setting

To ensure consistency with the referenced PIQE method, four widely used image databases, TID2013^35^, LIVE^36^, CSIQ^37^, and KADID-10k^40^ are employed for extensive evaluation. While PIQE originally used TID2008 as the evaluation database during its publication period, TID2013 is adopted in this study to enable broader comparisons than TID2008, making it the preferred choice for IQA. Database details are as follows: CSIQ: JPEG compression, JPEG-2000 compression, global contrast decrements, additive pink Gaussian noise, and Gaussian blurring. This resulted in 866 distorted versions of original images. TID2013: The database consists of six image sets; on average, 30 subjects have evaluated 12–14 devices depicting eight different scenes for a total of 79 different cameras, 480 images, and 188 subjects (67% female). LIVE: The Release 2 distortions include JPEG (169 images), JPEG2000 (175 images), white noise (145 images), Gaussian blur (145 images) and JPEG2000 with bit errors in simulated Rayleigh fading channel (145 images). KADID-10k contains 81 pristine images, each degraded by one of 25 distortions, 5 levels each. Each image is scored by 30 degradation category ratings.

Specifically, the PIQE method evaluates only four distortion types in the CSIQ database: JPEG2000 compression (JP2K), JPEG compression (JPEG), Gaussian blur (GB), and white noise (WN). To achieve a more comprehensive performance evaluation, TID2013 is employed with 24 distortion types, including additive Gaussian noise (AGN), Additive noise in color components is more intensive than additive noise in the luminance component (AGC), Spatially correlated noise (SCN), masked noise (MN), high-frequency noise (HFN), impulse noise (IN), quantization noise (QN), GB, image denoising (ID), JPEG compression, JPEG2000 compression, JPEG transmission errors (JPEGTR), JPEG2000 transmission errors (JP2KTR), non-eccentricity pattern noise (NEPN), local block-wise distortions (LBWD), mean shift (MS), contrast change (CC), change of color saturation (CCS), multiplicative Gaussian noise (MGN), comfort noise (CN), lossy compression of noisy images (LCNI), image color quantization with dither (LCQD), chromatic aberrations (CA), sparse sampling and reconstruction (SSR). All abbreviations are listed in Table 1 for reference and convenience.

To evaluate the prediction performance of the BIQA methods, two widely used metrics recommended by the video quality expert group^38^ are employed: Spearman rank order correlation coefficient (SROCC), which measures prediction monotonicity, and the Pearson linear correlation coefficient (PLCC), which measures prediction accuracy. Both metrics are reported for comparisons across the three selected databases, with higher values indicating better image quality estimation, unlike the PIQE score, where a lower value represents higher quality. A block size of 16 ⋅ 16 pixels is used as the basic processing unit, and all parameters and threshold values follow those defined in earlier equations. For a fair comparison, EPIQE is implemented based on MATLAB^39^ R2020b’s built-in PIQE code, ensuring consistency with the original method.

### Whole databases prediction performance comparison

This section presents a comprehensive comparison of the proposed EPIQE with PIQE and other MATLAB built-in IQA methods, including^41^, NIQE and BRISQUE^10^(Table 1). Since BRISQUE are supervised BIQA methods and have been extensively validated in prior studies, their results are also evaluated under the same databases and simulation settings to provide readers with a more meaningful comparison. Performance is assessed using SROCC and PLCC, with all results summarized in Table 1. For clarity, the highest-performing values for each metric across the methods are highlighted in bold at the bottom of the table without considering^41^ together for comparison.

In the CSIQ database, EPIQE achieves the highest SROCC and PLCC scores among all BIQA methods. Although its performance ranks second in the LIVE and TID2013 databases, the results are very close to the best-performing methods. For instance, in TID2013, EPIQE attained a PLCC of 0.8906, outperforming PIQE’s 0.8887 and approaching BRISQUE’s 0.8928 and NIQE’s 0.8812. The minimal difference of 0.0022 between BRISQUE and EPIQE is negligible, demonstrating EPIQE’s competitive capability in handling diverse distortion types and achieving results comparable to supervised BIQA methods. Overall, the experimental findings in Table 1 confirm that the proposed EPQIE method enhances PIQE’s effectiveness, offering a more comprehensive solution for IQA.

**Table 1:** SROCC and PLCC indices for prediction performance comparison on LIVE, CSIQ, TID2013 and KADID-10k databases.

Data base	Index	^41^	BRISQUE^10^	NIQE^12^	PIQE^30^	EPIQE
LIVE	SROCC	-	**0.9398**	0.9354	0.9370	0.9372
	PLCC	-	0.9675	0.9621	0.9761	**0.9768**
CSIQ	SROCC	0.9713	0.8520	0.9127	0.9454	**0.9486**
	PLCC	0.9833	0.9702	0.9759	0.9850	**0.9851**
TID 2013	SROCC	0.9736	0.7861	0.7846	0.7895	**0.7987**
	PLCC	0.9847	**0.8928**	0.8812	0.8887	0.8906
KADID-10k	SROCC	0.9283	**0.3980**	0.3380	0.2370	0.2391
	PLCC	0.9700	**0.4260**	0.3020	0.2890	0.2924

To further compare with recent research findings^41^, was used as the image quality estimation result after recent addition of neural network training. The data was obtained from the original paper^41^ and the “-” indicates that the original author did not perform this database test. It is evident that combining a large training database with a neural network yields image quality estimation results superior to rule of thumb. Nevertheless, this study maintains a simultaneous improvement in PIQE and the new database KADID-10k.

**Fig. 7:**
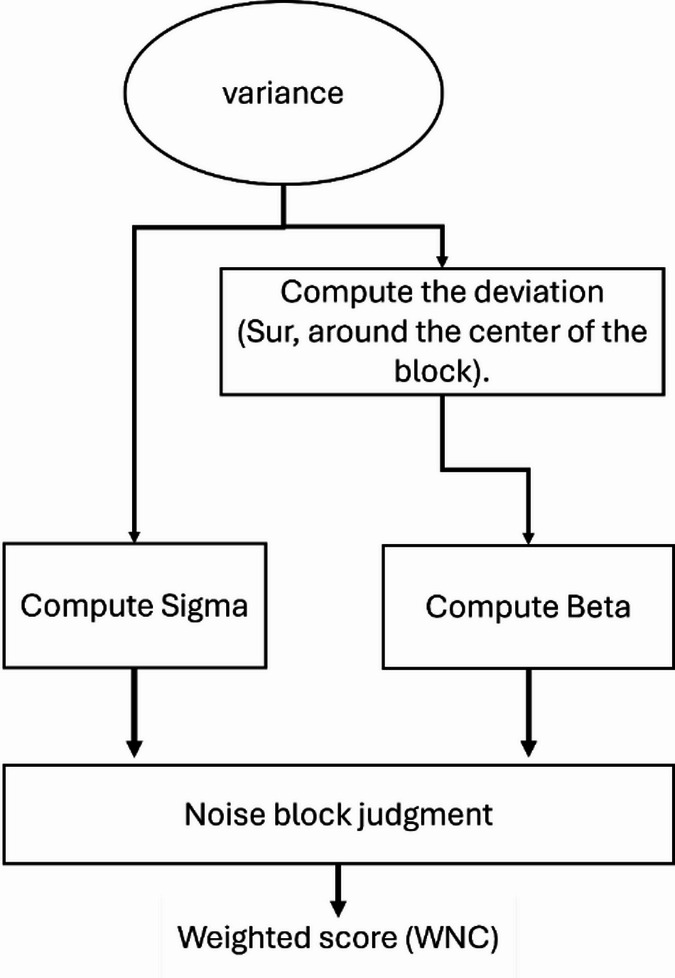
Statistical significance test results of the IQA model on datasets (**a**) LIVE, (**b**) CSIQ, (**c**) TID2013, and (**d**) KADID-10k. A green highlighted number “1” indicates that the model in that row is statistically superior to the model in that column, and a red highlighted number “0” indicates that the model in that row is statistically inferior to the model in the corresponding column. A “-” indicates that there is no statistically significant difference between the models in the corresponding row and column.

To obtain statistical conclusions regarding image quality assessment (IQA) performance, we further conducted a series of hypothesis tests, using methods similar to those described in reference^41^. The main objective was to determine whether the performance differences between the two IQA schemes were statistically significant. We performed hypothesis testing on the residuals between the DMOS values and the objective quality scores (after nonlinear mapping) provided by the IQA algorithm. Figure 7 shows the significance of test results on different IQA datasets. The number “1” indicates that the IQA method in that row is significantly better than the method in the column, and the number “0” indicates that the IQA method in that row is significantly worse than the method in the column. The symbol “-” indicates that there is no statistically significant difference between the two corresponding IQA methods.

When PIQE was published, it primarily used three databases (LIVE, CSIQ, and TID) for comparison. EPIQE’s main contribution was improving the estimation accuracy of the original PIQE. Adding KADID-10k as a test database is a crucial reference point, primarily used to examine whether this study can be effectively applied to the commonly used test databases. Experimental results show that EPIQE, compared to the original PIQE, still functions correctly on the KADID-10k database, indicating that the method used in this study can be applied to a wider range of natural imagery. While not an absolute advantage over the reference^41^, the results demonstrate its usability and effectiveness, which is a significant contribution to the study’s goal of improving PIQE estimation accuracy.

### Performance comparison on individual distortion types

It is evident that EPIQE consistently outperforms PIQE across all databases and evaluation indices. Furthermore, when compared with the supervised BIQA methods BRISQUE and NIQE, EPIQE demonstrates either the best or near-best prediction performance, as shown in the previous section.

For a broader comparison, the prediction capability of the four BIQA methods was evaluated across individual distortion types using SROCC as the performance metric. The analysis continues from the previous section, with the best performance for each method and distortion type highlighted in bold at the bottom of the table for easier reference. The abbreviation AVE represents the SROCC average of all distortion types for each BIQA method. EPIQE delivers the best results for AGN, GB, ID, JP2K, MS, CC, and LCQD (Table 2). In contrast, PIQE achieves the best performance in only two distortion types. Overall, EPIQE demonstrates a substantial improvement, securing top performance in seven distortion types. Although EPIQE is lower than PIQE for some distortion types (such as JPEGTR and JP2KTR), the degree is quite close. The main reason is that compression causes vertical and horizontal feature distortion, which makes the detection results judged as general images rather than vertical or horizontal fan-shaped features. Fortunately, the overall distortion type comparison is still higher than PIQE.

**Table 2:** SROCC values for individual distortion types in the TID2013 database.

Database	Distortion type	BRISQUE^10^	NIQE^12^	PIQE^30^	EPIQE
TID2013	AGN	**0.9840**	0.9800	0.9760	**0.9840**
	AGC	0.8360	**0.9320**	0.8760	0.8320
	SCN	0.9080	**0.9600**	0.7920	0.7860
	MN	0.8985	**0.9408**	0.9105	0.9025
	HFN	0.9600	**0.9720**	0.9360	0.9520
	IN	0.7880	**0.9960**	0.9920	0.9880
	QN	0.8440	**0.9560**	0.9520	0.9240
	GB	0.9760	0.9320	0.9800	**0.9840**
	ID	0.8146	0.7361	0.8790	**0.8870**
	JPEG	0.9160	**0.9760**	0.9600	0.9560
	JP2K	0.9150	0.8150	0.9452	**0.9635**
	JPEGTR	0.5440	**0.5760**	0.4440	0.5160
	JP2KTR	0.6600	**0.7600**	0.5000	0.4920
	NEPN	**0.6416**	0.4895	0.3622	0.3605
	LBWD	0.7705	0.6928	**0.8128**	0.8088
	MS	0.4040	0.4800	0.5880	**0.6240**
	CC	0.5762	0.5408	0.5745	**0.5869**
	CCS	**0.4840**	0.3440	0.3400	0.4080
	MGN	0.9790	**0.9830**	0.9510	0.9790
	CN	**0.6320**	0.5560	0.6000	0.6280
	LCNI	0.7680	**0.9440**	0.9080	0.9080
	LCQD	0.9438	0.9640	**0.9880**	**0.9880**
	CA	**0.8500**	0.7193	0.8090	0.8370
	SSR	**0.8960**	0.7600	0.8730	0.8609
	AVE	0.7912	0.7919	0.7895	0.7982

### Image quality score estimation improvement analysis on PIQE and EPIQE methods

**Fig. 8:**
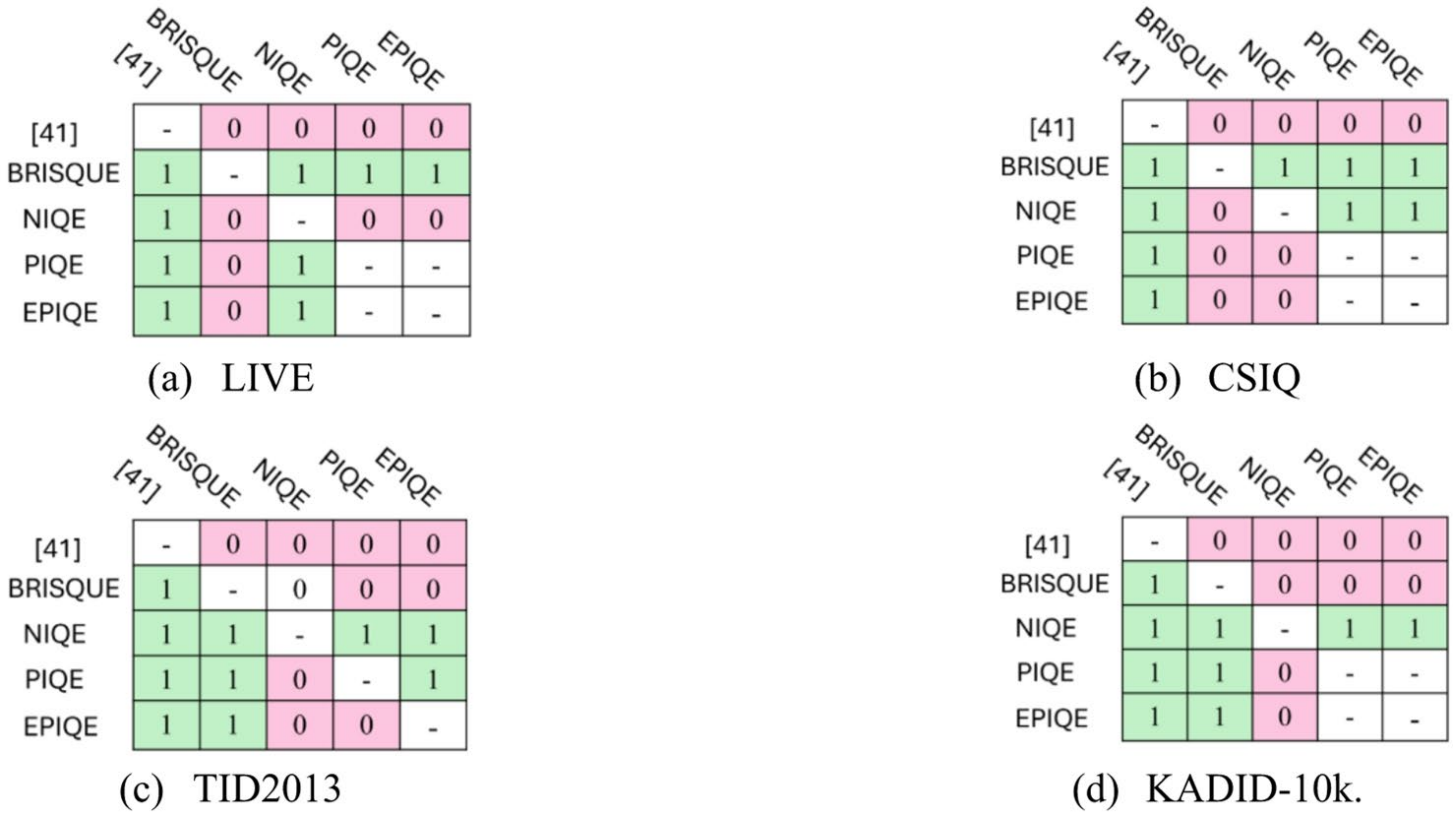
Four reference images from the TID2013 database with the highest PIQE scores.

**Fig. 9:**
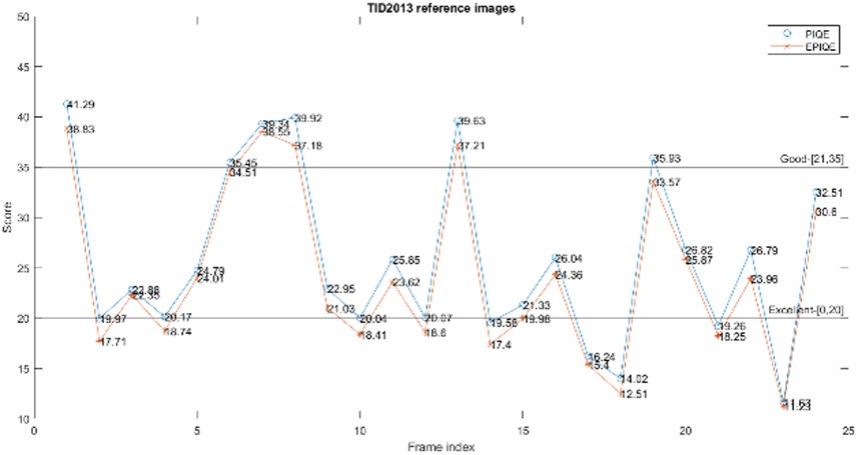
PIQE and EPIQE scores for each reference image in the TID2013 database.

Based on the original concept and the proposed methods, PIQE and EPIQE, developed under the same framework, their performance in real image quality score estimation was analyzed. In Sect. 2.2, for distortion-free and clean images from the TID2013 database, the expected ideal image quality should fall within the ‘Excellent’ or at least the ‘Good’ region. However, according to the PIQE-defined regions, up to six images were classified as being at the ‘Fair’ level, which is unacceptable for practical applications. For instance, in Fig. 8, four images are shown and clearly visualized, where their quality can easily be determined to be at least at a ‘Good’ level. This evaluation of 24 images from the TID2013 database presents their PIQE and EPIQE score values in Table 3, emphasizing two key aspects: image quality correction and enhancement levels. Across all 24 images, EPIQE consistently produces lower scores than PIQE, indicating correct image quality improvement. The estimated score trend is illustrated in Fig. 9. Regarding score correction, six images are reclassified from the ‘Good’ region to the ‘Excellent’ region, with scores improving as follows: I02 (19.97 to 14.84), I04 (20.07 to 16.63), I10 (20.04 to 16.89), I12 (20.07 to 17.37), I14 (19.58 to 14.96), and I21 (19.26 to 16.57). These results demonstrate that EPIQE estimates image quality more accurately than PIQE. Furthermore, clear improvements in enhancement degrees are shown in four images, with DIFF values of − 4.63 (I01), − 6.33 (I08), − 5.55 (I13), and − 4.39 (I19), due to the presence of significant horizontal fence-shaped content. In Table 3, DIFF represents the image quality score difference between PIQE and EPIQE, where a negative value “–” indicates prediction improvement, as a lower score corresponds to higher image quality in the PIQE definition.

The execution time, measured in seconds, is listed in Table 4. The evaluation was conducted on a personal computer with an Intel Core i7 1.8 GHz CPU and 16 GB RAM, and the average time was calculated using the TID2013 database. The results indicate that the enhanced noise feature criterion increases computational demand; however, this increase is justified by the substantial improvement in image quality estimation accuracy while maintaining controllable computational complexity. Using Gaussian filter parameters, vertical and horizontal detection is performed. The original MATLAB built-in function is not optimized and has a wide range of applications, which results in a much longer computation time than PIQE. In practical applications, the computation time can be shortened by rewriting and optimizing the function.

**Table 3:** SROCC comparison between PIQE and EPIQE on the TID2013 database with listed differences.

Database	Reference image number	PIQE^30^	EPIQE	DIFF
TID2013	I01	41.29	36.66	–4.63
	I02	**19.97**	**14.84**	–5.13
	I03	22.88	21.24	–1.64
	I04	**20.17**	**16.63**	–3.53
	I05	24.79	22.03	–2.75
	I06	**35.45**	**32.79**	–2.67
	I07	39.34	38.00	–1.34
	I08	39.92	33.60	–6.33
	I09	22.95	19.52	–3.43
	I10	**20.04**	**16.89**	–3.15
	I11	25.85	20.91	–4.95
	I12	**20.07**	**17.37**	–2.69
	I13	39.63	34.07	–5.55
	I14	**19.58**	**14.96**	–4.62
	I15	21.33	17.73	–3.61
	I16	26.04	23.67	–2.38
	I17	16.24	14.94	–1.30
	I18	14.02	9.48	–4.54
	I19	35.93	31.54	–4.39
	I20	26.82	24.66	–2.16
	I21	**19.26**	**16.57**	–2.69
	I22	26.79	20.99	–5.80
	I23	11.63	10.58	–1.05
	I24	32.51	28.06	–4.45

**Table 4:** Execution time comparison of four algorithms in seconds.

Index	BRISQUE^10^	NIQE^12^	PIQE^30^	EPIQE
Execution time	0.0071	0.0131	0.0247	0.1455

## Conclusion

This study focused on developing an NR BIQA method that eliminates training requirements while maintaining perceptual accuracy. The proposed EPIQE method preserves the strengths of PIQE while addressing its limitations, particularly the horizontal fence-shaped artifact, through a refined noise feature weighting strategy. To ensure broader applicability, four databases containing a wide range of distortion types were employed, and comparisons were conducted against PIQE, as well as^41^, BRISQUE and NIQE. All baseline methods were implemented using MATLAB built-in code, while EPIQE was developed within the PIQE framework for reproducibility and ease of deployment. Future work is expected to focus on recommending EPIQE into MATLAB^39^ and other widely used software platforms to establish it as a reliable evaluation tool, as training-free BIQA methods with strong predictive performance remain limited yet essential.

## Data Availability

The datasets used and/or analysed during the current study available from the corresponding author on reasonable request.
